# Interfacility Transfer of Uninsured vs Insured Patients With ST-Segment Elevation Myocardial Infarction in California

**DOI:** 10.1001/jamanetworkopen.2023.17831

**Published:** 2023-06-09

**Authors:** Michael J. Ward, Sayeh Nikpay, Andrew Shermeyer, Brahmajee K. Nallamothu, Ivan Rokos, Wesley H. Self, Renee Y. Hsia

**Affiliations:** 1 Department of Emergency Medicine, Vanderbilt University Medical Center, Nashville, Tennessee; 2 Department of Biomedical Informatics, Vanderbilt University Medical Center, Nashville, Tennessee; 3 Geriatric Research, Education, and Clinical Center, Tennessee Valley Healthcare System, Nashville, Tennessee; 4 Division of Health Policy and Management, University of Minnesota School of Public Health, Minneapolis; 5 Department of Internal Medicine, Division of Cardiovascular Medicine, University of Michigan, Ann Arbor; 6 Michigan Integrated Center for Health Analytics and Medical Prediction, Institute for Healthcare Policy and Innovation, University of Michigan, Ann Arbor; 7 Department of Emergency Medicine, UCLA-Olive View, Los Angeles, California; 8 Vanderbilt Institute for Clinical and Translational Research, Vanderbilt University Medical Center, Nashville, Tennessee; 9 Department of Emergency Medicine, University of California at San Francisco, San Francisco; 10 Philip R. Lee Institute for Health Policy Studies, University of California at San Francisco, San Francisco

## Abstract

**Importance:**

Insurance status has been associated with whether patients with ST-segment elevation myocardial infarction (STEMI) presenting to emergency departments are transferred to other facilities, but whether the facility’s percutaneous coronary intervention capabilities mediate this association is unknown.

**Objective:**

To examine whether uninsured patients with STEMI were more likely than patients with insurance to experience interfacility transfer.

**Design, Setting, and Participants:**

This observational cohort study compared patients with STEMI with and without insurance who presented to California emergency departments between January 1, 2010, and December 31, 2019, using the Patient Discharge Database and Emergency Department Discharge Database from the California Department of Health Care Access and Information. Statistical analyses were completed in April 2023.

**Exposures:**

Primary exposures were lack of insurance and facility percutaneous coronary intervention capabilities.

**Main Outcomes and Measures:**

The primary outcome was transfer status from the presenting emergency department of a percutaneous coronary intervention–capable hospital, defined as a facility performing 36 percutaneous coronary interventions per year. Multivariable logistic regression models with multiple robustness checks were performed to determine the association of insurance status with the odds of transfer.

**Results:**

This study included 135 358 patients with STEMI, of whom 32 841 patients (24.2%) were transferred (mean [SD] age, 64 [14] years; 10 100 women [30.8%]; 2542 Asian individuals [7.7%]; 2053 Black individuals [6.3%]; 8285 Hispanic individuals [25.2%]; 18 650 White individuals [56.8%]). After adjusting for time trends, patient factors, and transferring hospital characteristics (including percutaneous coronary intervention capabilities), patients who were uninsured had lower odds of experiencing interfacility transfer than those with insurance (adjusted odds ratio, 0.93; 95% CI, 0.88-0.98; *P* = .01).

**Conclusions and Relevance:**

After accounting for a facility’s percutaneous coronary intervention capabilities, lack of insurance was associated with lower odds of emergency department transfer for patients with STEMI. These findings warrant further investigation to understand the characteristics of facilities and outcomes for uninsured patients with STEMI.

## Key Points

**Question:**

Is insurance status associated with interfacility transfer among patients with ST-segment elevation myocardial infarction (STEMI) presenting to the emergency department?

**Findings:**

In this cohort study of 32 841 transferred patients with STEMI in California, uninsured patients had significantly lower odds of interfacility transfer compared with their insured counterparts regardless of the facility’s percutaneous coronary intervention capabilities.

**Meaning:**

These findings warrant further investigation to understand the characteristics of facilities and outcomes for uninsured patients with STEMI.

## Teaser

This cohort study examines whether uninsured patients with ST-segment elevation myocardial infarction (STEMI) were more likely than insured patients to experience interfacility transfer in California.

## Introduction

Management of ST-segment elevation myocardial infarction (STEMI) among the nearly 500 000 patients in the US who experience this condition annually requires timely access to reperfusion to optimize patient outcomes. Primary percutaneous coronary intervention (PCI) is the preferred strategy for emergent reperfusion^1,2^ and is recommended for patients with STEMI who are transferred in less than 120 minutes.^3,4^ Even though the proportion of patients with STEMI being transferred is increasing,^5^ regional variability in the availability of PCI further complicates efforts to deliver efficient and timely care, because 61% of hospitals lack PCI capabilities.^6^ In some regions, nearly one-half of patients with STEMI must be transferred to PCI centers.^7,8,9^ STEMI regionalization efforts such as the American Heart Association’s Mission: Lifeline sought to improve the timeliness and quality of care,^10^ and there has been increased access to PCI with improved quality of care and clinical outcomes over time in Mission: Lifeline hospitals.^11^

Although timely reperfusion with primary PCI is typically the primary justification for transfer, nonmedical factors associated with transfer may be potential sources of variation impacting optimal care delivery for this population. One nonmedical factor that has been previously explored is insurance status, specifically the lack of health insurance. Even though the ability to pay is prohibited as a screening criteria, as set forth by the Emergency Treatment and Labor Act of 1986,^12^ there is evidence that insurance status may impact transfer decision-making in emergency care settings.^13,14,15,16,17,18,19^ According to the Nationwide Emergency Department Sample, the largest sample of all-payer data sets for emergency department visits, uninsured emergency department visits with STEMI diagnostic codes were 60% more likely to be transferred than visits with any form of insurance.^5^ However, an important gap, and the focus of this work, is whether uninsured patients with STEMI simply present to systematically different facilities that do not have the capabilities to treat them (ie, PCI capabilities) and are, therefore, transferred.

This study sought to examine whether insurance status was associated with the odds of interfacility transfer for patients with STEMI presenting to the emergency department while accounting for a facility’s PCI capabilities. We hypothesized that without controlling for facility characteristics, uninsured patients with STEMI would have higher odds of interfacility transfer. However, we hypothesized that facility characteristics would act as a mediator between insurance status and transfer.

## Methods

This cohort study used nonpublic data from the California Health Care Access and Information (HCAI; formerly called the Office of Statewide Health Planning and Development) database, Patient Discharge Database, and the Emergency Department Discharge Database for 2010 to 2019. These data capture all hospital inpatient stays and emergency department visits at licensed, nonfederal, acute care hospitals in the state of California. This study was approved by the Vanderbilt University Medical Center institutional review board with a waiver of consent because this is secondary research and the identity of the participants cannot be readily identified, in accordance with 45 CFR §46. This study meets the criteria for reporting observational research defined in the Strengthening the Reporting of Observational Studies in Epidemiology (STROBE) reporting guideline.^20^

### Selection of Participants

We identified patients with STEMI using the following *International Classification of Diseases, Ninth Revision (ICD-9) *codes in the primary diagnostic impression: 410.00, 410.01, 410.10, 410.11, 410.20, 410.21, 410.30, 410.31, 410.40, 410.41, 410.50, 410.51, 410.60, 410.61, 410.80, 410.81, 410.90, and 410.91. We also used the following *International Statistical Classification of Diseases and Related Health Problems, Tenth Revision (ICD-10)* codes: I21.0, I21.01, I21.02, I21.09, I21.1, I21.11, I21.19, I21.2, I21.21, I21.29, and I21.3.^21,22,23,24^

### Exposures and Outcomes

The main exposure was lack of insurance. We used the primary reported payer category in the discharge records to assign patients to insurance status (eg, uninsured vs insured). Specifically, coverage from Medicare, Medi-Cal (California’s Medicaid program), a private (or commercial) insurer, other source (including workers’ compensation, county indigent programs, other indigent programs, other government programs, and other payers) were considered insured, and self-pay was categorized as uninsured. The primary outcome of our analysis was transfer status, defined as a discharge code reported as, “Discharged/transferred to a short-term general hospital for inpatient care.”

### Measurements

Recognizing that far fewer primary PCI procedures are performed for coronary reperfusion compared with PCIs in the nonemergent setting, we used annual counts of HCAI data on PCI volume to identify whether a facility could ever perform this procedure. Although we used PCI volume as a surrogate for a facility’s primary PCI capabilities, some facilities may not be able to treat patients with STEMI with primary PCI. PCI volume was identified using a total of 135 *ICD-9* and *ICD-10* codes (eTable 1 in Supplement 1). During the time frame of our study, the American College of Cardiology and American Heart Association jointly recommended that facilities without on-site cardiac surgery backup perform at least 36 primary PCIs annually to reduce door-to-balloon and in-hospital mortality.^25^ Thus, we categorized facilities with a threshold of 36 or more annual PCIs as meeting the definition of having PCI capabilities, whereas those with fewer than 36 PCIs did not. We treated this as a dichotomous variable in our results. However, recognizing that this threshold is for primary PCI rather than elective PCI, we implemented sensitivity analyses around it.

We created 3 groups of control variables from HCAI. The first group comprised patient characteristics. These include patient age, self-reported race and ethnicity prespecified by HCAI (American Indian, Asian, Black, Hispanic, White, or other [not defined by HCAI]), patient sex, the Elixhauser Comorbidity Index, and whether the patient presented to the transferring emergency department on the weekend. Race and ethnicity were included in this study because we wanted to adjust for differential treatment of racial and ethnic groups, such as structural or overt racism. We also included 2 patient-level characteristics measured at the level of the patient’s residence. These included rural status defined using the US Department of Agriculture Rural Urban Continuum Codes and the percentage of people in the county living below the Federal Poverty Level according to the Small Area Income and Poverty Estimates.^26^

The second group comprised facility-level characteristics, including whether the facility was located in a rural county (also assessed by US Department of Agriculture Rural Urban Continuum Codes 4-9), and hospital ownership, including nonprofit, for-profit, and public. We also included a categorical variable equal to the hospital’s quartile of patients with STEMI relative to all hospitals in California. The third group of controls consisted of a set of month-year indicator variables to control for nonlinear time trends.

From an original sample of 171 472 patients with STEMI observed between 2010 and 2019, we limited our sample to patients aged 18 years or older who were not missing demographic or geographic information and initial presentations of patients with STEMI to the emergency department. We sought to eliminate hospital stays from our sample in which a patient with STEMI had already been transferred. We did this by removing patients for whom the source of admission was another hospital. We also limited our sample to general acute care hospitals.

### Statistical Analysis

In our primary analyses, we assessed the association of insurance status (uninsured vs insured) with the odds of transfer for patients with STEMI using a multivariable logistic regression. Our regression included all patient-level and facility-level characteristics and month-year indicators described already. In secondary analyses, we conducted 3 stratified multivariable logistic regressions to assess the association of insurance status with the odds of transfer for key subgroups. First, we stratified according to whether the transferring hospital was capable of performing PCI. Second, we stratified according to whether the transferring hospital’s commercially insured patient with STEMI volume was in the top or bottom quartile of California hospitals. Finally, we stratified according to the transferring hospital’s ownership type. These subgroup analyses allowed us to assess whether the association of uninsured status with transfer status was similar across hospitals that could and could not perform PCI, across hospitals that serve more or fewer well-insured patients, and across hospitals that operate as nonprofits, for-profits, and public facilities. To account for common variation across patients over time, we clustered the SEs at the patient level. We present estimated odds ratios (ORs) and 95% CIs. Two-sided *P* < .05 was considered statistically significant. All analyses were performed using STATA/MP statistical software version 17.0 (StataCorp). Statistical analyses were completed in April 2023.

We conducted 4 sensitivity checks to determine whether the results were attributable to variable specification or our sample selection. The first consisted of redefining the uninsured variable using not just self-pay but also county indigent programs. This is because certain county-level programs, such as Healthy San Francisco,^27^ provide subsidized care at certain safety-net institutions but do not constitute insurance. The second involved limiting the sample to years after 2015, during which *ICD-10* diagnostic codes were used. This eliminated issues with respect to a change in codes used to define STEMI. The third consisted of limiting the analysis to 2013,^28^ in which survey data were linked to each facility in California to assess whether PCI capabilities were available 24 hours per day or only during certain hours in the day. The fourth excluded patients with Kaiser Permanente as a primary source of insurance. Because these insured patients are repatriated to a Kaiser Permanente facility for post-PCI hospital care, this may appear to be an interfacility transfer when it actually represents repatriation.

We conducted 2 exploratory analyses to control for treatment at the initial facility before interfacility transfer through receipt of PCI, thrombolytic therapy, or both before transfer among uninsured and insured patients. Therefore, for transferred patients, the hospital in which the patient appears was the one they were transferred from, and for patients who were not transferred the hospital in which they appeared was where they were ultimately treated. Finally, we ran a separate model for each quartile of annual PCI volume to examine how PCI capabilities impact the transfer of uninsured patients.

## Results

### Characteristics of Study Participants

**Figure 1.**
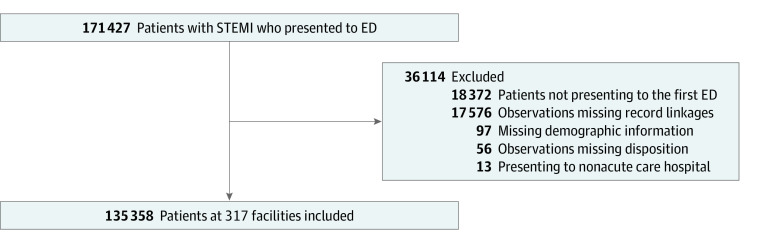
Participant Enrollment Flowchart ED indicates emergency department; and STEMI, ST-segment elevation myocardial infarction.

The final study population included 135 358 STEMI hospitalizations with 32 841 transferred patients (24.2%) (mean [SD] age, 64 [14] years; 10 100 women [30.8%]; 2542 Asian individuals [7.7%]; 2053 Black individuals [6.3%]; 8285 Hispanic individuals [25.2%]; 18 650 White individuals [56.8%]). This population included 120 150 unique patients and 317 unique hospitals (Figure 1). Additional characteristics of the patient population can be seen in Table 1. Compared with nontransferred patients, transferred patients were younger, were less likely to be female, had fewer Elixhauser comorbidity indices, and were more likely to report Hispanic race and ethnicity. The distribution of race differed significantly between transferred and nontransferred patients, although differences between categories were small. Transferred patients were also more likely to present on weekends, to live in rural counties, to present to a rural hospital that was also not PCI capable, and to present to public and for-profit than nonprofit hospitals. Transferred patients were also more likely to initially present to hospitals with fewer commercially insured patients with STEMI.

**Table 1.** Sample Characteristics for Patients With ST-Segment Elevation Myocardial Infarction by Transfer Status, 2010-2019

Characteristic	Patients, No. (%)		*P* value^a^
	Not transferred (n = 102 517)	Transferred (n = 32 841)	
Uninsured	5172 (5.1)	2150 (6.6)	<.001
Age, mean (SD), y	65 (14)	64 (14)	<.001
Sex			
Female	30 889 (30.1)	10 100 (30.8)	.03
Male	71 628 (69.9)	22 741 (69.2)	
Elixhauser Comorbidity Index, mean (SD)	0.66 (0.52)	0.63 (0.52)	<.001
Race and ethnicity			
American Indian	288 (0.3)	183 (0.6)	<.001
Asian	9721 (9.4)	2542 (7.7)	
Black	5707 (5.6)	2053 (6.3)	
Hispanic	20 988 (20.5)	8285 (25.2)	
White, non-Hispanic	60 806 (59.3)	18 650 (56.8)	
Other^b^	5007 (4.9)	1128 (3.4)	
Weekend presentation	2667 (2.6)	1803 (5.5)	<.001
Rural patient	2713 (2.7)	1984 (6.0)	<.001
Rural facility	875 (0.9)	1461 (4.5)	<.001
Percutaneous coronary intervention–capable hospital	89 551 (87.4)	8792 (26.8)	<.001
Annual emergency department volume, mean (SD), admissions	58 496 (26 367)	44 541 (25 680)	<.001
Commercial payment share quartile			
First	7102 (6.9)	6124 (18.6)	<.001
Fourth	27 061 (26.4)	7442 (22.7)	
Facility ownership			
Nonprofit	73 226 (71.4)	21 357 (65.0)	<.001
Public	12 476 (12.2)	5385 (16.4)	
For-profit	16 815 (16.4)	6099 (18.6)	

^a^ *P* values were calculated with a *t* test for the difference in population means and with a *z* test for the difference in population proportions between those who were or were not transferred.

^b^ Other race is not defined in the California Health Care Access and Information database.

**Figure 2.**
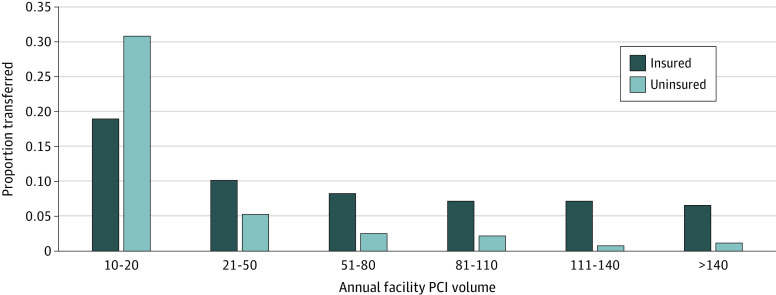
Proportion of Patients With ST-Segment Elevation Myocardial Infarction Transferred After Presenting to California Emergency Departments by Insurance Status and Facility Percutaneous Coronary Intervention (PCI) Volume, 2010-2019 Data are from the California 2010 to 2019 Department of Health Care Access and Information emergency and patient discharge databases.

Of the patients with STEMI identified in our sample, 102 517 were not transferred and 32 841 (24.2%) were transferred. Among the transferred patients, 2150 patients (6.6%) were uninsured, and the proportion of transferred uninsured patients was modestly higher than in the insured group (2150 of 7322 patients [29.4%] vs 30 691 of 128 036 patients [24.0%]). The proportion of transferred patients with STEMI by insurance status and annual facility PCI volume can be seen in Figure 2.

### Main Results

Before adding controls, the odds of transfer for uninsured patients with STEMI were 1.32 (95% CI, 1.25-1.39; *P* < .001) times the odds of transfer for insured patients with STEMI in California hospitals. After controlling for nonlinear secular time trends, the adjusted OR (aOR) was 1.35 (95% CI, 1.28-1.43; *P* < .001), and after controlling for patient-level demographics, health status, and geographic location, the aOR was 1.28 (95% CI, 1.21-1.35; *P* < .001). However, after controlling for characteristics of the transferring hospital in the final model (Table 2), the odds of transfer for uninsured patients were lower than the odds of transfer for insured patients (aOR 0.93; 95% CI, 0.88-0.98; *P* = .01).

**Table 2.** Main Model Results

Variable	Adjusted OR (95% CI)	*P* value
Uninsured	0.93 (0.88-0.98)	.01
Age	0.99 (0.99-0.99)	<.001
Sex		
Female	1 [Reference]	<.001
Male	0.95 (0.92-0.98)	
Year of presentation		
2010	1 [Reference]	NA
2011	1.12 (1.06-1.19)	<.001
2012	1.13 (1.06-1.19)	<.001
2013	1.17 (1.10-1.24)	<.001
2014	1.18 (1.10-1.24)	<.001
2015	1.24 (1.17-1.32)	<.001
2016	1.44 (1.35-1.53)	<.001
2017	1.28 (1.20-1.37)	<.001
2018	1.22 (1.14-1.31)	<.001
2019	1.17 (1.09-1.25)	<.001
Elixhauser Comorbidity Index	0.93 (0.91-0.96)	<.001
Race and ethnicity		
American Indian	1.09 (0.89-1.34)	.41
Asian	0.78 (0.74-0.82)	<.001
Black	0.97 (0.91-1.03)	.35
Hispanic	1.07 (1.03-1.11)	<.001
White non-Hispanic	1 [Reference]	NA
Other^a^	0.91 (0.84-0.98)	.02
Weekend presentation	1.68 (1.56-1.82)	<.001
Poverty	1.00 (1.00-1.00)	<.001
Rural patient	1.18 (1.09-1.29)	<.001
Percutaneous coronary intervention count annually	0.96 (0.96-0.96)	<.001
Emergency department volume mean	1.00 (1.00-1.00)	.01
Rural facility	1.06 (0.96-1.19)	.25
Commercial payment share quartile		
First	1 [Reference]	NA
Second	1.17 (1.12-1.23)	<.001
Third	0.90 (0.86-0.95)	<.001
Fourth	0.74 (0.71-0.78)	<.001
Facility ownership		
Nonprofit	1 [Reference]	NA
Public	0.98 (0.93-1.03)	.41
For-profit	0.93 (0.90-0.97)	.001

Abbreviations: NA, not applicable; OR, odds ratio.

^a^ Other race is not defined in the California Health Care Access and Information database.

### Sensitivity Analyses

The 4 robustness checks of underinsurance, *ICD-10* vintage, use of the 2013 PCI facility indicator, and exclusion of patients enrolled in Kaiser Permanente did not identify an increased odds of transfer for uninsured patients compared with insured patients (eTable 2 in Supplement 1). Next, after accounting for treatment at the initial facility with receipt of either PCI (aOR, 0.87; 95% CI, 0.82-0.93; *P* < .001) or fibrinolytics (aOR, 0.93; 95% CI, 0.88-0.98; *P* = .01), the adjusted odds of transfer for uninsured patients were lower than for insured patients if patients received either PCI or fibrinolytics (eTable 3 in Supplement 1). Finally, after accounting for transfer of uninsured patients stratified by quartile of annual hospital PCI volume, the odds of transfer for uninsured patients were no different than those for insured patients for the first quartile, and then were lower for the second through fourth quartiles (eTable 4 in Supplement 1). Notably, the OR of transfer for uninsured vs insured patients decreased across quartiles of annual PCI volume, indicating that the uninsured patients were less likely to be transferred in hospitals that did many PCIs per year compared with hospitals that did very few. In summary, we found that our results were not sensitive to the vintage of *ICD* code, the inclusion of underinsured individuals in our uninsured category, or facility annual PCI volume at the transferring hospital.

## Discussion

In this cohort study, we found that the transfer of patients with a time-sensitive emergency, STEMI, is highly dependent on the characteristics of the facility to which the patient initially presents. Using a definition of 36 PCI procedures per year in California, a state with one of the most regionalized STEMI networks in the US, we found that there were decreased odds of interfacility transfer of uninsured patients presenting to the emergency department. Multiple robustness checks confirmed that uninsured patients with STEMI had either lower or similar odds of transfer vs their insured counterparts.

Prior studies^13,14,15,16,17,18,19^ demonstrated a disproportionate increase in emergency interfacility transfers for uninsured patients. Unlike the motivating analysis on STEMI transfers,^29^ once we accounted for a facility’s PCI capability, an increased likelihood of transfer disappeared. This is consistent with facility characteristics being a mediator between insurance status and transfer status. A key advancement provided by this study is that after accounting for facility characteristics, including the PCI capabilities of those facilities, in our final model, the odds of transfer were now significant, but in the opposite direction. Our findings suggest that the facility’s characteristics, specifically PCI capabilities, are the primary factor associated with our current findings that uninsured patients had lower odds of transfer compared with their insured patients.

Although lack of insurance was not associated with higher odds of transfer, this does not mean that uninsured patients are consistently receiving the highest quality care. In preliminary analyses that warrant further investigation, we found that uninsured patients with STEMI were more likely to present to facilities without PCI capabilities.^30^ This finding suggests that uninsured individuals may have access to lower resourced facilities (ie, no PCI capabilities). However, for those uninsured patients presenting to lower volume PCI facilities, they have lower odds of staying at the facility compared with patients in facilities with the highest volume quartile (>85 annual PCI procedures). Because procedural volume can be an indicator of higher quality of care,^31,32^ uninsured patients may be more likely to access higher quality care provided at higher volume facilities, if they present to a facility with such capabilities.

A key factor that may contribute to the reduced odds of transfer for uninsured patients with STEMI from facilities with PCI capabilities is California’s robust STEMI regionalization, which was completed in 2014.^33^ Combined with early Medicaid expansion, regionalization was associated with reduced racial disparities in transfer rates and PCI after acute myocardial infarction.^34^ However, although regionalization enhanced access to PCI facilities, disparities in access, particularly among minoritized communities, remain in California.^23^ Combined with disproportionate presentation of uninsured patients to facilities without PCI capabilities and to those with lower volume facilities, these findings suggest that regionalization alone is not enough to enhance the quality and timeliness of care for socioeconomically disadvantaged populations. Important future directions for this work should examine the role of California’s regionalized STEMI network, whether patient outcomes are affected by transfer status, whether accounting for facility PCI capabilities in other states similarly mitigates uninsured transfer disparities, whether uninsured patients have access to different levels of quality of care, and how interfacility transfer may mitigate this potential quality gap.

### Limitations

Our results should be considered in light of several limitations. In addition to the inherent limitations associated with use of administrative data, our analyses were unable to examine clinical or situational factors at the time of patient presentation, such as the type of myocardial infarction, workload, and the actual PCI capabilities (ie, primary PCI) at the moment of transfer for the referring facilities. We also could not account for clinician-level factors that may impact the transfer decision, such as hemodynamic stability of the patient and clinical severity. Next, these results were from the California HCAI data set, which is from a state that expanded Medicaid before and during implementation of the Patient Protection and Affordable Care Act and may not be generalizable to other states and their respective patient populations. Within that population, there were only 7322 uninsured patients with STEMI, with 2150 transferred over a 10-year period. Furthermore, these data do not include the timeliness of transfers, nor does the transfer decision represent actual clinical outcomes for transferred patients; in some cases, these could be appropriate (in cases of transferring to a higher level of care from a non-PCI to PCI facility).

## Conclusions

Among patients with STEMI presenting to facilities with PCI capabilities, lack of insurance was associated with lower odds of interfacility transfer. Further work is needed to understand the generalizability of these findings and whether access to high-quality care remains an important barrier for uninsured patient outcomes.

## Notes

Supplement 1. **eTable 1.** *ICD-9* and *ICD-10* PCI Procedure Codes to Identify Facility PCI and Fibrinolytic Capabilities **eTable 2.** Sensitivity Tests for the Primary Model **eTable 3.** Exploration of Treatment at the Transferring Facility **eTable 4.** Exploration of Annual PCI Volume at the Transferring Facility Among PCI Capable Facilities, by Quartile

Supplement 2.
Data Sharing Statement
