# Error in Methods Section

**DOI:** 10.1001/jamanetworkopen.2023.26207

**Published:** 2023-07-14

In the Original Investigation titled “Interfacility Transfer of Uninsured vs Insured Patients With ST-Segment Elevation Myocardial Infarction in California,”^1^ published June 9, 2023, the Rural Urban Continuum Codes shown in the Methods section were incorrect (codes 4-9 should have been codes 7-9). This article has been corrected.^1^
