# Supplementary material for: Interfacility Transfer of Uninsured vs Insured Patients With ST-Segment Elevation Myocardial Infarction in California
Source: JAMA Netw Open. 2023 Jun 9;6(6):e2317831. doi: 10.1001/jamanetworkopen.2023.17831 (PMC10257096; doi:10.1001/jamanetworkopen.2023.17831)
Supplement: Supplement 1. — eTable 1. ICD-9 and ICD-10 PCI Procedure Codes to Identify Facility PCI and Fibrinolytic Capabilities eTable 2. Sensitivity Tests for the Primary Model eTable 3. Exploration of Treatment at the Transferring Facility eTable 4. Exploration of Annual PCI Volume at the Transferring Facility Among PCI Capable Facilities, by Quartile [file jamanetwopen-e2317831-s001.pdf]

Supplemental Online Content

Ward MJ, Nikpay S, Shermeyer A, et al. Interfacility transfer of uninsured vs insured patients with ST-segment elevation myocardial infarction in California. *JAMA Netw Open*. 2023;6(6):e2317831. doi:10.1001/jamanetworkopen.2023.17831

eTable 1. *ICD-9* and *ICD-10* PCI Procedure Codes to Identify Facility PCI and Fibrinolytic Capabilities
eTable 2. Sensitivity Tests for the Primary Model
eTable 3. Exploration of Treatment at the Transferring Facility
eTable 4. Exploration of Annual PCI Volume at the Transferring Facility Among PCI Capable Facilities, by Quartile

This supplemental material has been provided by the authors to give readers additional information about their work.

© 2023 Ward MJ et al. *JAMA Network Open.*

eTable 1. *ICD-9* and *ICD-10* PCI Procedure Codes to Identify Facility PCI and Fibrinolytic Capabilities

	ICD-9	ICD-10	ICD-10 Suffixes
PCI	0.66	02703	46, 4Z
	17.55	02704	56, 5Z
	36.00	02713	66 ,6Z
	36.04	02714	76, 7Z
	36.06	02723	D6, DZ
	36.07	02724	E6, EZ
	36.09	02733	FZ, GZ
		02734	Z6, ZZ
Thrombolytics	39.65	5A1522F	
	99.10	3E03317	
		3E03017	
		3E04317	
		3E04017	
		3E06317	
		3E06017	

© 2023 Ward MJ et al. *JAMA Network Open.*

eTable 2. Sensitivity Tests for the Primary Model

	Check 1: Underinsured + Uninsured (N=135,358)				Check 2: ICD10 Years Only (N=43,641)				Check 3: Only 2013 Facility PCI Coding (N=15,235)				Check 4: Exclude Kaiser Patients (N=119,855)			
	Adjusted Odds Ratio	95% CI			Adjusted Odds Ratio	95% CI			Adjusted Odds Ratio	95% CI			Adjusted Odds Ratio	95% CI		
Uninsured	0.81	0.77	-	0.85	0.94	0.83	-	1.07	0.89	0.76	-	1.04	1.03	0.96	-	1.10
Year of Presentation (ref=2010)			-		-		-		-		-		-		-	
2011	1.12	1.06	-	1.19	-		-		-		-		1.16	1.08	-	1.25
2012	1.12	1.06	-	1.19	-		-		-		-		1.14	1.06	-	1.22
2013	1.17	1.10	-	1.24	-		-		-		-		1.22	1.13	-	1.31
2014	1.16	1.09	-	1.23	-		-		-		-		1.22	1.13	-	1.31
2015	1.23	1.15	-	1.31	-		-		-		-		1.30	1.21	-	1.40
2016	1.43	1.34	-	1.52	-		-		-		-		1.53	1.42	-	1.66
2017	1.27	1.18	-	1.36	0.90	0.84	-	0.96	-		-		1.41	1.30	-	1.53
2018	1.21	1.12	-	1.29	0.84	0.78	-	0.91	-		-		1.39	1.27	-	1.51
2019	1.15	1.07	-	1.23	0.80	0.74	-	0.86	-		-		1.37	1.25	-	1.49
Age	0.99	0.99	-	0.99	1.00	1.00	-	1.01	0.99	0.99	-	0.99	0.99	0.99	-	0.99
Sex (ref=Female)	0.94	0.91	-	0.97	1.05	0.99	-	1.11	0.94	0.84	-	1.04	0.93	0.89	-	0.97
Elixhauser Comorbidity Index	0.90	0.87	-	0.93	0.78	0.74	-	0.82	0.96	0.88	-	1.05	0.89	0.86	-	0.92
Patient Race (Ref=Non-Latinx White)																
Asian	0.97	0.91	-	1.04	1.16	1.04	-	1.29	1.01	0.81	-	1.26	0.89	0.82	-	0.96
Black	1.09	0.89	-	1.34	1.40	1.03	-	1.90	0.85	0.48	-	1.52	1.14	0.90	-	1.44
Latinx	0.78	0.74	-	0.82	0.85	0.78	-	0.92	0.80	0.68	-	0.94	0.83	0.78	-	0.88
Native American	0.91	0.84	-	0.98	1.10	0.97	-	1.25	1.08	0.85	-	1.38	0.84	0.76	-	0.92
Other	1.07	1.03	-	1.11	1.17	1.10	-	1.25	1.04	0.93	-	1.17	1.08	1.03	-	1.13
Poverty	1.00	1.00	-	1.00	1.00	1.00	-	1.00	1.00	1.00	-	1.00	1.00	1.00	-	1.00
Weekend Presentation	1.69	1.56	-	1.83	1.64	1.52	-	1.78	-		-		1.73	1.57	-	1.90
Rural Patient	1.19	1.09	-	1.29	1.21	1.04	-	1.41	1.12	0.89	-	1.42	1.31	1.19	-	1.45
PCI Count Annually	0.96	0.96	-	0.96	0.96	0.96	-	0.96	-		-		0.94	0.94	-	0.94
2013 PCI Indicator	-		-		-		-		0.25	0.22	-	0.29	-		-	
ER Volume Mean	1.00	1.00	-	1.00	1.00	1.00	-	1.00	1.00	1.00	-	1.00	1.00	1.00	-	1.00
Ownership (ref=Non-Profit)																
Public	0.99	0.94	-	1.03	0.92	0.85	-	1.01	0.87	0.75	-	1.00	0.96	0.91	-	1.02
For-Profit	0.94	0.90	-	0.97	0.94	0.87	-	1.00	0.88	0.77	-	1.00	0.91	0.87	-	0.95
Commercial Payment Share Quartile (ref=1st)																
2nd	1.17	1.12	-	1.23	1.27	1.17	-	1.38	0.99	0.85	-	1.15	1.26	1.20	-	1.33
3rd	0.90	0.86	-	0.95	0.91	0.83	-	0.99	1.06	0.91	-	1.23	1.04	0.98	-	1.10
4th	0.74	0.70	-	0.78	0.96	0.87	-	1.05	0.55	0.46	-	0.65	0.83	0.78	-	0.89
Rural Facility	1.06	0.96	-	1.18	1.16	0.92	-	1.46	-		-		0.82	0.73	-	0.92

Note: The table presents the results of specification tests of our preferred logistic regression. Model 1 includes both under-insured and uninsured in the uninsured indicator. Model 2 limits the sample to 2016 and 2017 to only use only ICD-10 codes for STEMI. Model 3 presents data only from 2013 using validated, survey-based data on PCI capability. Model 4 excludes patients with Kaiser as a primary source of insurance.

© 2023 Ward MJ et al. *JAMA Network Open.*

eTable 3. Exploration of Treatment at the Transferring Facility

	**Check 1: Received PCI Before Transfer (N=135,358)**		**Check 2: Received Fibrinolytics Before Transfer (N=135,358)**	
	**Adjusted Odds Ratio**	**95% CI**	**Adjusted Odds Ratio**	**95% CI**
Uninsured	0.87	0.82 - 0.93	0.93	0.88 - 0.98
Year of Presentation (ref=2010)	-	-	-	-
2011	1.13	1.07 - 1.20	1.12	1.06 - 1.19
2012	1.16	1.09 - 1.24	1.12	1.06 - 1.19
2013	1.23	1.15 - 1.31	1.17	1.10 - 1.24
2014	1.25	1.17 - 1.33	1.17	1.10 - 1.24
2015	1.45	1.36 - 1.55	1.24	1.17 - 1.32
2016	1.73	1.62 - 1.85	1.44	1.35 - 1.54
2017	1.67	1.56 - 1.80	1.28	1.20 - 1.37
2018	1.69	1.57 - 1.82	1.22	1.14 - 1.31
2019	1.64	1.52 - 1.77	1.17	1.09 - 1.26
Age	0.99	0.99 - 0.99	0.99	0.99 - 0.99
Sex (ref=Female)	0.86	0.83 - 0.89	0.95	0.92 - 0.98
Elixhauser Comorbidity Index	0.91	0.88 - 0.94	0.90	0.88 - 0.93
Patient Race (Ref=Non-Latinx White)				
Asian	0.90	0.84 - 0.97	0.97	0.91 - 1.03
Black	0.97	0.78 - 1.20	1.09	0.89 - 1.34
Latinx	0.80	0.76 - 0.84	0.78	0.74 - 0.82
Native American	0.97	0.89 - 1.05	0.91	0.84 - 0.98
Other	1.05	1.01 - 1.09	1.07	1.03 - 1.11
Poverty	1.00	1.00 - 1.00	1.00	1.00 - 1.00
Weekend Presentation	1.48	1.36 - 1.61	1.69	1.56 - 1.83
Rural Patient	1.09	1.00 - 1.18	1.19	1.09 - 1.29
PCI Count Annually	0.98	0.97 - 0.98	0.96	0.96 - 0.96
ER Volume Mean	1.00	1.00 - 1.00	1.00	1.00 - 1.00
Ownership (ref=Non-Profit)				
Public	0.97	0.92 - 1.02	0.98	0.93 - 1.03
For-Profit	0.96	0.92 - 1.00	0.93	0.90 - 0.97
Commercial Payment Share Quartile (ref=1st)				
2nd	1.19	1.14 - 1.26	1.17	1.12 - 1.23
3rd	0.92	0.87 - 0.97	0.90	0.86 - 0.95
4th	0.75	0.71 - 0.79	0.74	0.71 - 0.78
Rural Facility	1.03	0.92 - 1.16	1.06	0.96 - 1.19
Treatment Prior to Transfer				
PCI	0.20	0.19 - 0.21	-	-
Fibrinolytics	-	-	1.01	0.91 - 1.13

© 2023 Ward MJ et al. *JAMA Network Open.*

eTable 4. Exploration of Annual PCI Volume at the Transferring Facility Among PCI Capable Facilities, by Quartile

	Annual PCI Volume															
	1st Quartile				2nd Quartile				3rd Quartile				4th Quartile			
	Adjusted Odds Ratio		95% CI		Adjusted Odds Ratio		95% CI		Adjusted Odds Ratio		95% CI		Adjusted Odds Ratio		95% CI	
Uninsured	0.97	0.88	-	1.07	0.73	0.60	-	0.88	0.28	0.20	-	0.40	0.28	0.19	-	0.43
Year of Presentation (ref=2010)																
2011	1.22	1.12	-	1.34	1.18	1.00	-	1.39	0.90	0.74	-	1.08	0.93	0.77	-	1.14
2012	1.22	1.11	-	1.33	1.16	0.99	-	1.38	1.05	0.87	-	1.26	1.04	0.86	-	1.26
2013	1.31	1.19	-	1.44	1.33	1.13	-	1.56	0.97	0.81	-	1.16	1.31	1.09	-	1.59
2014	1.38	1.26	-	1.52	1.25	1.06	-	1.48	1.07	0.89	-	1.29	1.15	0.95	-	1.38
2015	1.48	1.35	-	1.63	1.32	1.10	-	1.58	1.26	1.04	-	1.52	1.67	1.37	-	2.04
2016	1.82	1.65	-	2.02	1.55	1.30	-	1.84	1.36	1.13	-	1.64	1.80	1.48	-	2.19
2017	2.27	2.01	-	2.55	1.44	1.20	-	1.74	1.55	1.25	-	1.91	1.38	1.09	-	1.74
2018	2.94	2.54	-	3.41	1.33	1.12	-	1.58	1.49	1.21	-	1.83	1.53	1.24	-	1.90
2019	3.14	2.68	-	3.69	1.69	1.44	-	1.99	1.37	1.10	-	1.70	1.28	1.02	-	1.62
Age	0.98	0.98	-	0.98	1.00	1.00	-	1.00	1.01	1.00	-	1.01	1.01	1.01	-	1.01
Sex (ref=Female)	0.85	0.81	-	0.89	0.93	0.85	-	1.01	0.93	0.84	-	1.02	0.93	0.84	-	1.03
Elixhauser Comorbidity Index	0.93	0.89	-	0.98	0.88	0.82	-	0.95	0.92	0.85	-	1.00	0.89	0.81	-	0.97
Patient Race (Ref=Non-Latinx White)																
Asian	0.72	0.66	-	0.80	1.24	1.06	-	1.46	1.67	1.42	-	1.96	0.95	0.77	-	1.18
Black	1.00	0.73	-	1.36	0.38	0.12	-	1.17	0.92	0.37	-	2.30	0.39	0.06	-	2.71
Latinx	0.88	0.80	-	0.97	0.85	0.75	-	0.96	1.03	0.89	-	1.18	1.14	0.95	-	1.36
Native American	0.97	0.83	-	1.13	1.00	0.84	-	1.19	1.15	0.96	-	1.38	1.14	0.94	-	1.37
Other	0.99	0.93	-	1.06	1.32	1.21	-	1.45	1.23	1.10	-	1.36	1.05	0.93	-	1.18
Poverty	1.00	1.00	-	1.00	1.00	1.00	-	1.00	1.00	1.00	-	1.00	1.00	1.00	-	1.00
Weekend Presentation	1.26	1.09	-	1.46	1.62	1.32	-	1.99	1.26	0.99	-	1.61	1.61	1.20	-	2.15
Rural Patient	1.20	1.04	-	1.39	0.78	0.55	-	1.10	0.39	0.25	-	0.61	0.60	0.43	-	0.85
PCI Count Annually	-		-		0.98	0.98	-	0.98	1.00	0.99	-	1.00	1.00	1.00	-	1.00
ER Volume Mean	1.00	1.00	-	1.00	1.00	1.00	-	1.00	1.00	1.00	-	1.00	1.00	1.00	-	1.00
Ownership (ref=Non-Profit)															-	
Public	0.70	0.65	-	0.75	1.80	1.62	-	2.01	0.66	0.55	-	0.80	0.57	0.47	-	0.68
For-Profit	0.86	0.80	-	0.92	0.99	0.90	-	1.10	1.05	0.94	-	1.18	0.98	0.84	-	1.15
Commercial Payment Share Quartile (ref=1st)																
2nd	1.41	1.31	-	1.52	1.24	1.10	-	1.40	0.46	0.38	-	0.56	0.77	0.30	-	1.95
3rd	1.27	1.18	-	1.38	0.89	0.78	-	1.03	0.31	0.26	-	0.38	0.72	0.28	-	1.84
4th	0.67	0.62	-	0.73	1.03	0.90	-	1.10	0.36	0.30	-	0.44	0.63	0.25	-	1.62
Rural Facility	0.75	0.64	-	0.88	0.36	0.21	-	0.62	-		-		-		-	

Note: The results depict estimated adjusted odds ratios from the full model for quartiles for sub-samples of our analytic sample: hospitals performing between 0 and 10 PCIs per year, 11-53 per year, 54-85 per year, and finally above 85 per year.

© 2023 Ward MJ et al. *JAMA Network Open.*
