# Supplementary material for: Interfacility Transfer of Uninsured vs Insured Patients With ST-Segment Elevation Myocardial Infarction in California
Source: JAMA Netw Open. 2023 Jun 9;6(6):e2317831. doi: 10.1001/jamanetworkopen.2023.17831 (PMC10257096; doi:10.1001/jamanetworkopen.2023.17831)
Supplement: Supplement 2. — Data Sharing Statement [file jamanetwopen-e2317831-s002.pdf]

Data Sharing Statement

Ward. Interfacility Transfer of Uninsured vs Insured Patients With ST-Segment Elevation Myocardial Infarction in California. *JAMA Netw Open*. Published June 09, 2023. doi:10.1001/jamanetworkopen.2023.17831

Data
Data available: No
